# Exploiting the Feedstock Flexibility of the Emergent Synthetic Biology Chassis *Vibrio natriegens* for Engineered Natural Product Production

**DOI:** 10.3390/md17120679

**Published:** 2019-11-30

**Authors:** Gregory A. Ellis, Tanya Tschirhart, Joseph Spangler, Scott A. Walper, Igor L. Medintz, Gary J. Vora

**Affiliations:** 1 Center for Bio/Molecular Science and Engineering, U.S. Naval Research Laboratory, Washington, DC 20375, USA; gregory.ellis@nrl.navy.mil (G.A.E.); scott.walper@nrl.navy.mil (S.A.W.); igor.medintz@nrl.navy.mil (I.L.M.); 2 American Society for Engineering Education, Postdoctoral Research Associate, U.S. Naval Research Laboratory, Washington, DC 20375, USA; 3 National Academy of Sciences, National Research Council, Postdoctoral Research Associate, U.S. Naval Research Laboratory, Washington, DC 20375, USA; joseph.spangler.ctr@nrl.navy.mil

**Keywords:** *Vibrio*, beta-carotene, violacein, marine bacteria

## Abstract

A recent goal of synthetic biology has been to identify new chassis that provide benefits lacking in model organisms. *Vibrio natriegens* is a marine Gram-negative bacterium which is an emergent synthetic biology chassis with inherent benefits: An extremely fast growth rate, genetic tractability, and the ability to grow on a variety of carbon sources (“feedstock flexibility”). Given these inherent benefits, we sought to determine its potential to heterologously produce natural products, and chose beta-carotene and violacein as test cases. For beta-carotene production, we expressed the beta-carotene biosynthetic pathway from the sister marine bacterium *Vibrio campbellii*, as well as the mevalonate biosynthetic pathway from the Gram-positive bacterium *Lactobacillus acidophilus* to improve precursor abundance. Violacein was produced by expressing a biosynthetic gene cluster derived from *Chromobacterium violaceum*. Not only was *V. natriegens* able to heterologously produce these compounds in rich media, illustrating its promise as a new chassis for small molecule drug production, but it also did so in minimal media using a variety of feedstocks. The ability for *V. natriegens* to produce natural products with multiple industrially-relevant feedstocks argues for continued investigations into the production of more complex natural products in this chassis.

## 1. Introduction

In order to expand the capabilities of engineered organisms, synthetic biologists have begun exploring non-model organisms that display characteristics not found in model organisms such as *Escherichia coli* [1,2,3]. The type strain of *Vibrio natriegens* is a Gram-negative, facultatively anaerobic, salt marsh isolate [4], that has been put forward as an emergent synthetic biology chassis due in large part to its non-pathogenic nature and extremely fast growth rate (reported doubling time < 10 min) [5,6,7,8,9,10,11,12]. To put this in context, its growth rate using glucose is at least two times faster than some industrially-relevant microorganisms including *E. coli*, *Bacillus subtilis*, *Corynebacterium glutamicum*, and yeast [7]. *V. natriegens* has a growth rate in glucose minimal medium (1.7 h^−1^) that is higher than even *E. coli* that has undergone adaptive laboratory evolution (~1.0 h^−1^), and 1.0 h^−1^ appears to be the upper limit for *E. coli* growth rate [9].

Many natural characteristics of *V. natriegens* support the investigation of this emergent chassis for bioproduction use in particular. First, *V. natriegens* has been characterized phenotypically and genomically, and is believed to be a generally safe biological agent (i.e., BSL-1) [5,7,8,9,10,11,12]. Second, as *V. natriegens* has garnered interest as an emergent chassis, genetic tools have been developed [8,10,12]. In concert with this, its extremely fast growth rate assists in genetically engineering this organism [7,12,13]. Third, its tolerance to high salt—in some media, up to 30 g·L^−1^ (~0.51 M) with an optimum at 15 g·L^−1^ (~0.26 M)—coupled to fast growth could help maintain axenic fermentation conditions without expensive antibiotic techniques; high salt tolerance has been referenced as an advantage for other emergent chassis such as *Halomonas* which grows in 60 g·L^−1^ NaCl [7,14,15]. Illustrating the flexibility of *V. natriegens*, if non-corrosive fermenters are not available to accommodate high-salt growth, it can still be grown with a low chlorine-based media [7,14,15]. Fourth, to support its rapid growth, *V. natriegens* must intrinsically be able to consume carbon sources very efficiently. Indeed, in regards to glucose, it has been found to have a remarkably high biomass specific substrate consumption rate (q_s_) of greater than double that of *E. coli*, *Pseudomonas putida*, yeast, and *C. glutamicum* (20X for *C. glutamicum*) and an uptake rate of 3.90 g g^−1^·h^−1^ (21.4 mmol g_DW_^−1^ h^−1^) [7,9]. This portends the ability for *V. natriegens* to likewise efficiently convert carbon sources to bioproducts [7]. Finally, and of particular importance in this study, is that *V. natriegens* is special in that it can grow efficiently on a variety of industrially-relevant feedstocks [7]. In fact, vibrios dedicate approximately half of their open reading frames to metabolism (carbohydrates, RNA, and proteins) [8]. Feedstock flexibility is incredibly important for consideration of an emergent chassis for bioproduction, as at large scales, the feasibility of a given bioprocess can be influenced by the cost, availability, and competing uses of the carbon source [16]. In fact, a major cost factor for microbial bioproduction of bulk chemicals is the carbon source [17,18]. The ability of chassis to grow and produce on a variety of feedstocks allows for adjusting to market changes in feedstock prices. Further, since most hydrolysates are a mixture of carbon sources, co-utilization of these breakdown products is extremely desirable; unfortunately, this natural ability is rare in microbial production chassis [16].

Inspired by these inherent *V. natriegens* characteristics, we sought to investigate its ability to heterologously produce natural products as example bioproduction targets and chose the carotenoid beta-carotene and the bis-indole violacein as target cases. Both of these natural products have previously been heterologously-expressed in microbial chassis [19,20,21,22,23,24,25,26,27,28,29]; our goal here was not to compete with these other chassis (e.g., for production yield), but to demonstrate the ability of *V. natriegens*, which has desirable intrinsic characteristics and potential feedstock flexibility, as an alternative chassis for natural product production.

## 2. Results

### 2.1. Investigating *V. natriegens* Feedstock Flexibility

To investigate the ability of *V. natriegens* to grow on diverse substrates, *V. natriegens* ATCC 14048 was subjected to Phenotype MicroArray™ testing using OmniLog^®^ V. 1.5 Comparison (BioLog, Hayward, CA; see Materials and Methods for more information). Cells were grown in proprietary minimal media in a microplate with individual wells supplemented by different carbohydrate sources, and cell respiration was determined kinetically by measuring NADH production through reduction of a tetrazolium dye over time. The average response height for each well throughout the assay was normalized to a reference cell without substrate, and those carbohydrates allowing cellular respiration over a threshold level of 50 are depicted in Table 1 (See Appendix A, Table A1, for additional information on screened carbon sources). While these results indicate respiration and substrates that may be more metabolically favorable, this does not necessarily correlate to growth rate. However, these results do corroborate and extend upon the findings by Hoffart et al. and Tschirhart et al. who examined *V. natriegens* growth rates with a variety of carbon sources [7,10]. In fact, all carbohydrates shown by Hoffart et al. and Tschirhart et al. to allow *V. natriegens* growth were found above the response threshold in our screen as well (Hoffart et al. also demonstrated *V. natriegens* grew with starch, which was not tested in our screen) [7,10].

Based upon these findings and their potential as feedstocks, we chose seven carbon sources to investigate (glucose, fructose, arabinose, glycerol, mannitol, N-acetyl-glucosamine, or sucrose at 4 g·L^−1^, 0.4%) in minimal medium along with LBv2 rich medium for production of natural products (Table 1). In terms of growth as determined by Hoffart et al., the carbon sources can be broken into two groups: Sucrose, N-acetyl glucosamine, glucose, and fructose promote fast growth (>1.5 h^−1^); while glycerol and arabinose promote slower growth (<1 h^−1^) (mannitol was not tested by Hoffart et al., but in Tschirhart et al. had a doubling time closer to the carbon sources promoting fast growth) [7,10]. 

### 2.2. Heterologous Production of Beta-carotene in *V. natriegens*

#### 2.2.1. Construction and Expression of The *V. Campbellii* Beta-Carotene Biosynthesis Pathway and *L. acidophilus* Mevalonate Pathway

**Figure 1:**
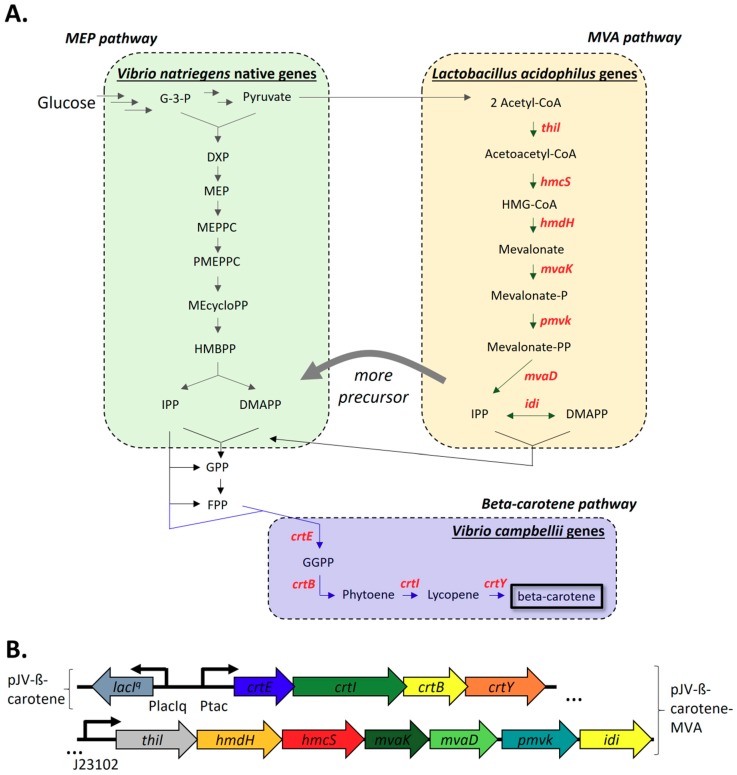
Summarized natural and heterologous pathways for beta-carotene production investigated herein. (**A**). *V. natriegens* naturally encodes the MEP (2-*C*-methyl-d-erythritol-4-phosphate) pathway (green, left). The *L. acidophilus* MVA (mevalonate) pathway was heterologously-expressed to provide more IPP/DMAPP precursor (yellow, right). These precursors eventually fed into the heterologously-expressed *V. campbellii* beta-carotene biosynthesis pathway (blue, bottom). Heterologously-expressed genes (by common/heterologous names) are in bold red; see Appendix B, Table A2 for gene IDs and enzyme names. (**B**). Layout of plasmids pJV-ß-carotene (top) and pJV-ß-carotene-MVA (top plus bottom). Beta-carotene biosynthetic genes overlap. Abbreviations: G-3-P, _D_-glyceraldehyde-3-phosphate; DXP, 1-deoxy-_D_-xylulose-5-phosphate; MEP, 2-C-methyl-d-erythritol-4-phosphate; MEPPC, 4-(cytidine 5’-diphospho)-2-C-methyl-d-erythritol; PMEPPC, 2-phospho-4-(cytidine 5’-diphospho)-2-C-methyl-d-erythritol; MEcycloPP, 2-C-methyl-d-erythritol 2,4-cyclodiphosphate; HMBPP, 1-hydroxy-2-methyl-2-butenyl 4-diphosphate; IPP, isopentenyl diphosphate; DMAPP, dimethylallyl diphosphate; GPP, geranyl diphosphate; FPP, farnesyl diphosphate; GGPP, geranylgeranyl diphosphate; HMG-CoA, 3-hydroxy-3-methylglutaryl-CoA; MVA, mevalonate [24,26,27,28,29,30,31].

**Figure A1:**
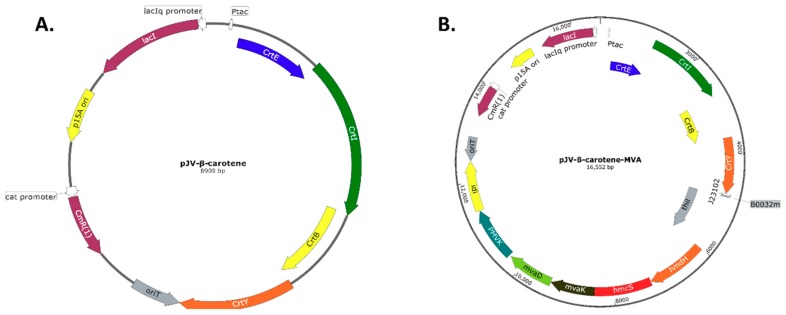
Plasmid maps for beta-carotene synthesis. A. Plasmid pJV-ß-carotene. B. Plasmid pJV-ß-carotene-MVA. Abbreviations: lacI^q^, *lac* repressor with mutation to diminish leaky expression; CmR, chloramphenicol resistance gene; all biosynthetic genes listed in text and Table A2 in Appendix B.

While *V. natriegens* does not naturally encode the four key biosynthetic genes necessary for beta-carotene production, it does contain one upstream pathway to produce the two key building blocks for isoprenoids: Isopentenyl pyrophosphate (IPP) and dimethylallyl pyrophosphate (DMAPP). This pathway is termed the 2-*C*-methyl-d-erythritol-4-phosphate (MEP) pathway, and branches from glycolysis, starting with glyceraldehyde-3-phosphate and pyruvate, as seen in Figure 1A [8,11,30,31]. We chose four genes for beta-carotene synthesis that utilize these building blocks from the sister species *V. campbellii* BAA-1116 (Figure 1A; see Appendix B, Table A2 for gene IDs and enzyme names) [32,33]. This cluster was placed under the control of an isopropyl-ß-D-thiogalactoside (IPTG)-inducible P_tac_ promoter (plasmid pJV-ß-carotene) (Figure 1A,B; for plasmid map, see Appendix C, Figure A1A) [34,35]. 

Extending beyond heterologously-expressing intragenus genes, and to potentially improve the production of the IPP and DMAPP precursors, we also expressed the mevalonate pathway from *Lactobacillus acidophilus* NCFM [36]. This pathway has been used similarly before in other bacteria to increase the yield of beta-carotene [24,26,27,28,29]. The mevalonate pathway branches from pyruvate at the end of glycolysis as seen in Figure 1A. Together, six enzymes convert two acetyl-CoA molecules to IPP. IPP can then be isomerized to DMAPP by the action of the seventh enzyme isopentenyl pyrophosphate isomerase (which can also help balance IPP/DMAPP from the first pathway to meet beta-carotene production needs). The genes were cloned with constitutive expression into the same plasmid as the beta-carotene genes from *V. campbellii* (plasmid pJV-ß-carotene-MVA) (Figure 1B; for plasmid map, see Appendix C, Figure A1B). This brought the total to 11 genes (in a ~16 kb plasmid) that were heterologously-expressed in this emergent chassis. 

#### 2.2.2. Heterologous Production of Beta-Carotene in Nutrient Rich Medium

**Figure 2:**
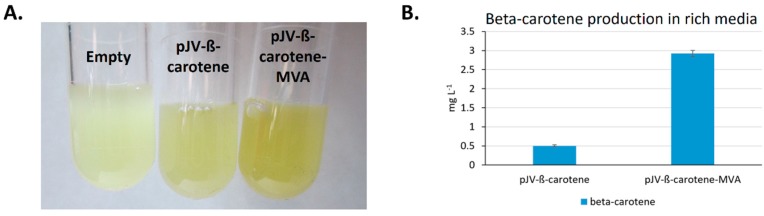
Beta-carotene production in LBv2 rich medium. (**A**). Visual analysis of beta-carotene production as the presence of more orange color by induced, transformed *V. natriegens* indicated no production with empty vector (left) but moderate and considerable production with pJV-ß-carotene (center) and pJV-ß-carotene-MVA (right). (**B**). HPLC-UV analysis at λ = 453 nm indicated that addition of the MVA pathway significantly increased production almost 6-fold (*p* < 0.001).

We first tested production of beta-carotene in LBv2 rich medium before exploring the different carbon sources in minimal media. Using only the four beta-carotene biosynthetic genes and the endogenous MEP pathway (pJV-ß-carotene), *V. natriegens* was able to produce 0.50 ± 0.02 mg·L^−1^ of beta-carotene as determined by HPLC-UV. This production increased significantly (*p* < 0.001) almost 6-fold to 2.93 ± 0.08 mg·L^−1^ when the additional mevalonate pathway was also expressed (plasmid pJV-ß-carotene-MVA) as shown in Figure 2.

#### 2.2.3. Feedstock Flexibility of *V. natriegens* for Producing Beta-carotene

**Figure 3:**
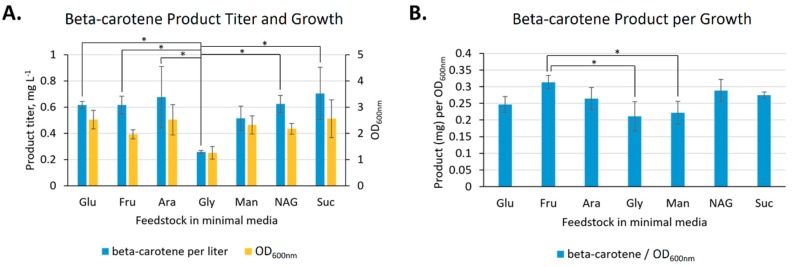
Feedstock flexibility of beta-carotene production. (**A**). *V. natriegens* containing pJV-ß-carotene-MVA produced beta-carotene (blue) in each carbon source (0.4% w/v) tested in minimal medium. The pattern of production roughly followed growth (yellow). (**B**). Beta-carotene production per growth. Abbreviations: Glu, glucose; Fru, fructose; Ara, arabinose; Gly, glycerol; Man, mannitol; NAG, N-acetyl glucosamine; Suc, sucrose; * statistically-significant differences for beta-carotene product titer and product per OD_600nm_ (α = 0.05) while statistically-significant differences in growth are not indicated to ease interpretation of graphs.

We next sought to investigate the feedstock flexibility of *V. natriegens* for beta-carotene production. *V. natriegens* harboring the pJV-ß-carotene-MVA plasmid was grown overnight in minimal media supplemented with additional NaCl, MOPS, and 4 g·L^−1^ (0.4%) of each carbon source. Final relative growth of the transformed *V. natriegens* in the different carbon sources followed the trends seen in Hoffart et al. and Tschirhart et al., with sucrose, glucose, fructose, mannitol, and N-acetyl-glucosamine promoting greater growth and glycerol promoting lesser growth (Figure 3A). One noticeable difference was arabinose, which also promoted high growth here; however, herein (1) we measured end-point growth and not growth rate, and (2) the expression of the beta-carotene pathway and resistance cassette could have affected growth. 

The relative production of beta-carotene on each carbon source largely followed the growth patterns (Figure 3A,B). In terms of overall production, nearly all carbon sources produced similar amounts of beta-carotene. Glycerol was the exception, producing significantly less than glucose, fructose, arabinose, N-acetyl glucosamine, or sucrose (α = 0.05) and this was perhaps due in some cases to significantly lower *V. natriegens* growth in glycerol versus glucose, arabinose, and sucrose (α = 0.05). On a production per growth (OD_600nm_) basis, all carbon sources performed similarly (with the exception of growth in fructose producing significantly more than glycerol or mannitol, α = 0.05). This bodes well for the use of *V. natriegens* as a bioproduction chassis with feedstock flexibility (see Discussion).

### 2.3. Heterologous Production of Violacein in V. natriegens

#### 2.3.1. Construction of the *C. violaceum* Violacein Biosynthesis Pathway *in V. natriegens*

**Figure 4:**
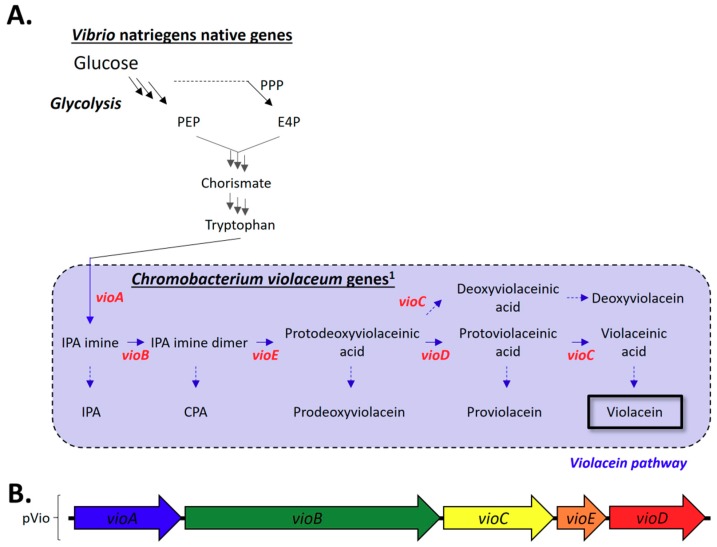
Natural and heterologous pathways for violacein. (**A**). *V. natriegens* naturally produces tryptophan from phosphoenolpyruvate (PEP) from glycolysis and erythrose-4-phosphate (E4P) from the pentose phosphate pathway. Five genes (bold red) were heterologously expressed to produce violacein from tryptophan; see Appendix B, Table A2, for gene IDs and enzyme names. Note that multiple arrows indicate multiple steps. (**B**). Layout of plasmid pVio. Additional abbreviations: IPA, indole pyruvic acid; CPA, chromopyrrolic acid. ^1^ Note that genes from *C. violaceum* were previously recoded for *E. coli* [8,11,37,42,43,44].

**Figure A2:**
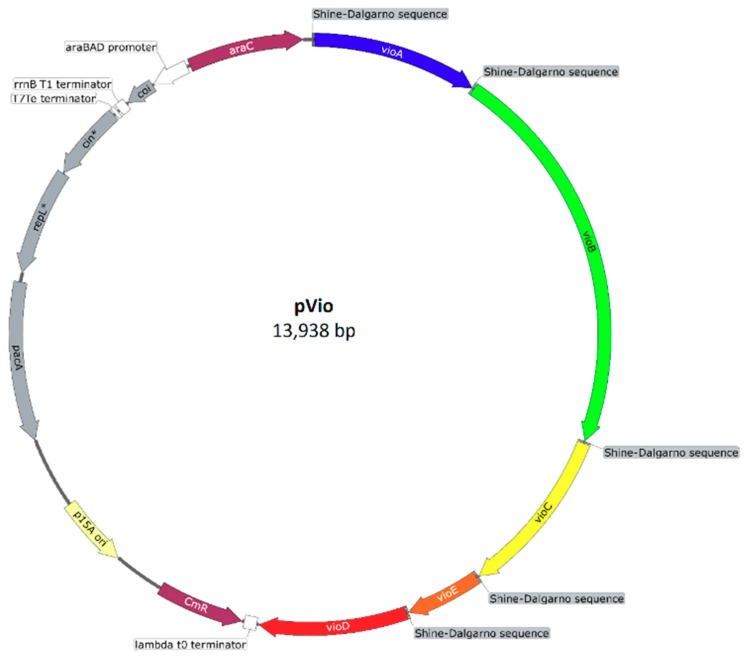
Plasmid map for violacein synthesis: Plasmid pVio. Abbreviations: CmR, chloramphenicol resistance gene; all biosynthetic genes listed in text and Table A2 in Appendix B.

To expand upon our investigation of heterologous natural product production in *V. natriegens*, we chose to explore the production of violacein. *V. natriegens* also does not naturally encode for production of violacein but does encode for the precursor tryptophan [8,11,37]. Five enzymes are responsible for transforming tryptophan to violacein; the violacein pathway also contains numerous opportunities for non-enzymatic conversion of intermediates to terminal co-products, which can limit overall yield, as seen in Figure 4A (see Appendix B, Table A2 for gene IDs and enzyme names) [22,37,38,39,40,41]. For violacein production, a plasmid (pVio) containing the violacein biosynthetic pathway (*vioABEDC* from *Chromobacterium violaceum* ATCC 12472) was used (Figure 4B; for plasmid map see Appendix C, Figure A2).

#### 2.3.2. Heterologous Production and Feedstock Flexibility of Violacein Production

**Figure 5:**
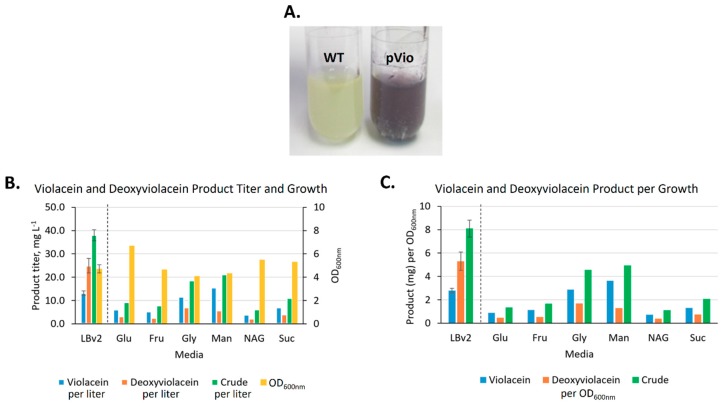
Violacein production in rich medium and minimal media + various carbon sources. (**A**). Visual analysis of violacein/deoxyviolacein production by *V. natriegens* indicated no production with WT (left) but considerable production with *V. natriegens* transformed with pVio (right). (**B**). *V. natriegens* containing pVio produced violacein (blue) and deoxyviolacein (orange) in LBv2 rich medium and each carbon source (0.4% w/v) tested in minimal media. The pattern of production did not follow growth (yellow). See text for explanation regarding arabinose testing. (**C**). Violacein production per OD_600nm_. Abbreviations: crude, summed violacein and deoxyviolacein; Glu, glucose; Fru, fructose; Gly, glycerol; Man, mannitol; NAG, N-acetyl glucosamine; Suc, sucrose; dashed line, separation between rich and minimal media

We tested *V. natriegens* containing the pVio plasmid in both LBv2 rich medium as well as the minimal media containing different carbon sources described above for production of deoxyviolacein (byproduct) and violacein. *V. natriegens* was able to produce 24.9 ± 3.1 mg·L^−1^ of deoxyviolacein and 13.1 ± 0.9 mg·L^−1^ of violacein in rich medium as determined by HPLC-UV (Figure 5).

Interestingly, the carbon sources that yielded the greatest production of beta-carotene did not yield the greatest production of violacein (Figure 5B,C). In particular, growth in mannitol produced more violacein than glucose, fructose, or N-acetyl-glucosamine (more than glucose or N-acetyl-glucosamine on a per OD_600nm_ basis). In fact, the minimal medium + mannitol (15.5 mg·L^−1^) and LBv2 rich medium both produced a similar amount of violacein. While the use of mannitol also resulted in higher production of beta-carotene as well, it was not substantially higher than the production observed from use of the other carbon sources. Growth in glycerol also appeared to produce high levels of violacein while its use resulted in a low level of beta-carotene production. Also interesting was the ratio of deoxyviolacein production versus violacein production: In rich medium, more deoxyviolacein was produced relative to violacein (~1.9X), whereas in minimal medium supplemented with the different carbon sources, more violacein was produced relative to deoxyviolacein (~4.2X, on average). Another notable observation was that violacein production by minimal medium + arabinose was excluded since the results would likely be confounded by the arabinose-induced production of the phagemid elements in the plasmid that is used (pVio) [43,44]. In fact, growth of the pVio-containing transformant in minimal medium + arabinose was lower than with the other carbon sources, potentially indicating cellular stress due to the production of these proteins or, less likely, an activation of prophages that resulted in the lysis of *V. natriegens* [45].

## 3. Discussion

*V. natriegens* intrinsically has many desirable characteristics for a bioproduction chassis: Fast growth rate, genetic tractability, biological safety, and growth on a wide variety of carbon sources [4,5,6,7,8,9,10,11,12,13,46]. In regards to growth rate in particular, it has been shown that expression of some heterologous genes can decrease expression of others, due to competition for finite resources in the cell such as RNA polymerase and ribosomes [47,48]. One could expect that fast growth would require more demand on these finite resources, which would then be less available for heterologous expression and a liability for bioproduction. However, it has been shown that increasing growth rate in *V. natriegens* increases ribosome number up to an estimated 115,000 ribosomes per cell, higher than the estimated 70,000 in *E. coli* (estimated ~90,000 if *E. coli* could grow as fast as *V. natriegens*). The greater number of ribosomes per cell may therefore mitigate some of this liability [46,49]. We also anticipate that *V. natriegens* will be able to utilize carbon sources very efficiently (*vide supra*). In this study, we took advantage of the ability of *V. natriegens* to utilize a variety of feedstocks and examined the production of two natural products by *V. natriegens* using a complex nutrient rich medium and minimal media supplemented with one of seven carbon sources: glucose, fructose, arabinose, glycerol, mannitol, and N-acetyl-glucosamine, and sucrose.

The carbon sources were chosen based on their natural metabolism by *V. natriegens* and their potential or current use for industrial bioproduction. Most bioproduction is based on glucose from molasses or from starch hydrolysis [16,18]. Fructose can be derived from sucrose hydrolysis or from wood, but typically costs more than glucose [50,51,52]. Arabinose is found in hemicellulose; *E. coli* can use this sugar but is limited by glucose repression, whereas natural *S. cerevisiae* strains cannot use this sugar [16]. Glycerol is easily available as it is a stoichiometric by-product of biodiesel production [16]. Mannitol is one of the most abundant sugars in brown macroalgae (also abundant are carbohydrate polymers alginate and glucan) [53]. Brown macroalgae as a feedstock source is advantageous as it requires no arable land, fresh water, or fertilizer; it is produced in many countries; it is easier to refine due to a lack of lignin; and other products (e.g., proteins) can be used from it as well [53]. N-acetyl glucosamine constitutes chitin; chitin is one of the most abundant polymers in nature, can be retrieved as waste from the shrimp industry, and is considered an under-utilized feedstock [16]. Finally, sucrose from sugarcane and sugar beet is a particularly desirable feedstock, as it is economical and environmentally friendly [54,55]. In fact, it is predicted to become the cheapest feedstock [55,56], in part because cane juice or molasses can be directly fermented (or, if need be, sucrose can be easily purified). In contrast, to utilize glucose, starch must be milled and hydrolyzed [18,57,58]. Further, after sucrose extraction, the remaining sugarcane biomass (bagasse) can be burned for fermentation processes; however, glucose-based processes require fossil fuel burning [18,59]. Importantly, many industrial *E. coli* strains cannot naturally use sucrose without engineering, whereas *V. natriegens* can [5,7,8,54,56,57].

### 3.1. Heterologous Production of Beta-carotene

Carotenoids such as beta-carotene have multiple uses industrially, including for health and as a pigment [60,61]. In addition to beta-carotene as a target chemical, partially overlapping isoprenoid pathways produce other valuable chemicals such as isoprene, arteminisin, limonene, and paclitaxel (Taxol); demonstration of beta-carotene production helps open the door to these compounds as well [27,62,63,64,65,66,67]. Sans optimization, which was beyond the scope of this effort, we noted that the production of beta-carotene in *V. natriegens* grown in rich medium (0.5 mg·L^−1^) is on par with production in *E. coli* (~ 1 mg·L^−1^) [26,60]. The inclusion of the MVA synthetic pathway, while improving production by ~6-fold, did not improve it to the levels seen in *E. coli* [26]. We anticipate that enhanced production may be possible by balancing the MVA pathway to preclude buildup of intermediates [68] or through other methods as discussed below. In terms of feedstock flexibility in minimal media, *V. natriegens* was found to produce beta-carotene in all seven carbon sources tested. We demonstrated that production of beta-carotene roughly followed the relative growth on each carbon source. This may be expected, as the substrates for beta-carotene production (IPP and DMAPP) are biosynthesized from central metabolism glycolysis intermediates/products which would also support growth. Of particular interest was that beta-carotene was produced relatively well in sucrose, portending the use of this desirable feedstock for production of natural products in this chassis. 

### 3.2. Heterologous Production of Violacein

In addition to beta-carotene, we also chose to target violacein, a bacterially-produced bis-indole with reported antibacterial, antitumoral, antiviral, trypanocidal, antiprotozoan, and antioxidant activities [19,20]. While the bacteria *Chromobacterium violaceum*, *Duganella* sp., and *Janthinobacterium lividum* naturally produce violacein, some of the titers (0.85–1.62 g·L^−1^), their propensity for forming non-violacein-producing variants in culture, and for *C. violaceum* and *J. lividum* their potential pathogenicity, limit their use for the industrial production of violacein [20]. Our efforts demonstrated that *V. natriegens* heterologously produced violacein (and deoxyviolacein) in both rich media and in minimal media containing the six carbon sources tested. In rich medium, the production of violacein with *V. natriegens* (~13 mg·L^−1^) was similar to the production observed in *E. coli* (~15 mg·L^−1^) when using a plasmid containing the same RBS as pVio [21], although higher production has been demonstrated in other optimized systems [23,25,69,70]. Interestingly, production was high when grown in mannitol or glycerol. High production of violacein with glycerol has also been noted before, though the reasons are unclear [70]. We speculate that the higher degree of reductance for glycerol (4.67) and mannitol (4.33) than for the other carbon sources (4) may increase the concentrations of NAD(P)H in the cell, a needed co-factor for VioD and VioC (we note, however, that we did not notice a significant increase in beta-carotene production for growth in these carbon sources although NADH is needed for its production as well) [60,71,72]. The high production in mannitol portends the ability to produce violacein from brown macroalgae, especially if *V. natriegens* can be engineered to utilize alginate; its ability to naturally use mannitol lessens the burden of engineering that aspect as well. Another interesting finding was that more deoxyviolacein was produced in rich media relative to violacein, whereas more violacein was produced in the supplemental minimal media relative to deoxyviolacein. This could be due to inhibition or relatively lesser production of VioD in rich media.

In conclusion, we demonstrate for the first time that *V. natriegens* is capable of substantial heterologous production of the natural products beta-carotene and violacein using a variety of carbon sources and that this production required the use of some of the largest plasmids (up to 16.5 kb) that have been successfully inserted and maintained in this bacterium. The heterologous multi-gene pathways introduced here were between 4.5–12 kb and consisted of up to 11 genes. These pathways, cloned from three different bacteria, did not need to be codon optimized for expression in *V. natriegens*. Overall, this first demonstration of heterologous natural product production using these different carbon sources serves to further develop the biotechnological usefulness of this organism, which has also been investigated for production of alanine, poly-ß-hydroxybutyrate, and selenium nanoparticles, all using native genes [5,7,73]. We anticipate that these results will encourage future efforts at optimizing bioproduction of other complex natural products in this chassis. 

## 4. Materials and Methods

### 4.1. Materials and Nomenclature

For all isolations of DNA using polymerase chain reaction (PCR) amplification, and for all PCR amplifications prior to Gibson assembly, Q5 High-Fidelity Polymerase or Q5 Hot Start High-Fidelity Polymerase was used (New England Biolabs, Ipswich, MA, USA) to minimize mutations. Colony PCR was done using NEB’s Taq polymerase (New England Biolabs, Ipswich, MA, USA). For all DNA preparations, either the Monarch^®^ Plasmid Miniprep Kit (New England Biolabs, Ipswich, MA, USA) or the QIAprep^®^ Spin Miniprep kit was used (Qiagen, Venlo, Netherlands). DNA cleaning and/or concentrating was done using the DNA Clean and Concentrator^®^-5 kit (Zymo Research, Tustin, CA, USA) as needed between PCR/amplification steps, unless otherwise specified. Gibson assembly was done using the Gibson Assembly^®^ Cloning Kit (New England Biolabs, Ipswich, MA, USA) with conditions found with NEBuilder (https://nebuilder.neb.com/, New England Biolabs, Ipswich, MA, USA). All sequencing was performed through Eurofins Genomics (Eurofins Genomics, Luxembourg, Luxembourg). Restriction digestion enzymes came from New England Biolabs (New England Biolabs, Ipswich, MA, USA). All chemicals, including carbon sources, were from Sigma-Aldrich (Sigma-Aldrich, St. Louis, MO, USA), Fisher Scientific (Thermo Fisher Scientific, Waltham, MA, USA), EMD (MilliporeSigma, Burlington, MA, USA), or Cayman Chemicals (Cayman Chemical Company, Ann Arbor, MI, USA). LBv2 medium was made up of 1x LB medium (Miller) with v2 salts. V2 salts consist of: 204 mM NaCl, 4.2 mM KCl, and 23.14 mM MgCl_2_ [12]. For the minimal medium base we used a variation of M9 minimal salts medium with added NaCl (48 mM Na_2_HPO_4_, 22 mM KH_2_PO_4_, 18.7 mM NH_4_Cl, 209 mM NaCl, 0.1 mM CaCl_2_, 2 mM MgSO_4_, 100 mM MOPS, 0.4% carbon source per L). Gene names used are either as specified on the National Center for Biotechnology Information website (NBCI; ncbi.nlm.nih.gov) or common homologous names [74,75,76,77]. *V. natriegens* ATCC 14048 was the strain type used in all final transformations and production studies. Pathways were investigated with help of KEGG (Kanehisa laboratories https://www.kegg.jp). Genome information used was from Bethesda (MD): National Library of Medicine (US), NCBI, and for *V. natriegens* was from accession numbers NZ_CP009977.1 and NZ_CP016345.1, for *V. campbellii* from accession numbers NC_009783.1 and NC_022269.1, for *L. acidophilus* from accession number NC_006814.3, and for *C. violaceum* from accession number NC_005085.1 [74,75,76,77,78]. For a list of plasmids used in this study, see Table 2.

### 4.2. BioLog Phenotype MicroArrays™

*V. natriegens* ATCC 14048 was submitted to BioLog (Hayward, CA, USA) for Phenotype MicroArrays (PMs) for Microbial Cells^TM^ analyses on the OmniLog system^®^. Cells were grown on Biolog Universal Growth media with sheep’s blood at 30 °C overnight. They were then added into the wells of the plates, which were run according to a proprietary protocol for running Gram-negative bacteria. The base test media for the metabolic test plates is proprietary, but it is a minimal media composed mostly of salt and buffers. For more information, see the BioLog website (https://www.biolog.com/products-portfolio-overview/phenotype-microarrays-for-microbial-cells/) and reference papers [79,80].

### 4.3. Cloning of the Beta-Carotene Pathway

The four gene beta-carotene biosynthetic cluster (see Appendix B, Table A2) was PCR amplified from *V. campbellii* ATCC BAA-1116 genomic DNA using primers 1 and 2 (Table 3), digested with Dpn1, and underwent A-tailing. A-tailing was accomplished using Taq polymerase and following a protocol from New England Biolabs, but substituting in standard Taq buffer and a dNTP mix (New England Biolabs, Ipswich, MA). The A-tailed construct was inserted into a pCR™4-TOPO^®^ TA vector using the TOPO™ TA Cloning Kit™ for Sequencing (Thermo Fisher Scientific, Waltham, MA, USA) and the reaction transformed into Top10 *E. coli*. DNA preparation afforded the plasmid TOPO-ß-carotene. 

The beta-carotene cluster was then inserted into the pJV298 plasmid [35] by Gibson assembly. Primers 3 and 4 were used to PCR amplify the beta-carotene cluster from plasmid TOPO-ßcar and primers 5 and 6 were used to PCR amplify pJV298. Reactions were digested with DpnI, and assembled with Gibson assembly. Assembly reactions were transformed into *E. coli* NEB10β, and DNA preparation afforded the plasmid pJV-beta-carotene. The insertion of the ß-carotene cluster was confirmed by colony PCR (using primers 1 and 2), patch plating confirming bacteria produced more orange pigment on plates containing IPTG, plasmid restriction digestion (HindIII), and sequencing. Plasmid pJV-ß-carotene was then electroporated into *V. natriegens* as described in Tschirhart et al. [10], resulting in strain *V. natriegens* pJV-ß-carotene. For a list of DNA features used for the different plasmids, see Table 4.

### 4.4. Cloning of the MVA Pathway

The two clusters (MVA1 = *thil*, *hmdH*, *hmcS*; MVA2 = *mvaK*, *mvaD*, *pmvk*, *idi* (Appendix B, Table A2) were PCR amplified separately from *L. acidophilus* NCFM genomic DNA [81] using primers 7 and 8, and 9 and 10, respectively. MVA1 underwent A-tailing, was inserted into the pCR™4-TOPO^®^ TA vector similarly to above, and the reaction transformed into TOP10 *E. coli*. DNA preparation afforded the plasmid TOPO-MVA1. MVA2 was inserted into the pCR™4Blunt-TOPO^®^ vector using the TOPO™ Blunt-End Cloning Kit™ for Sequencing (Thermo Fisher Scientific, Waltham, MA, USA) and the reaction transformed into TOP10 *E. coli*. DNA preparation afforded the plasmid TOPO-MVA2.

**Table 2:** Plasmids used in this study.

#	Name	Features	Refs.
1	pJV298	oriT, p15A ori, lacIq, *gfp* under lacIq control, CmR	[35]
2	pA5D5GFP	J23102 promoter, B0032 RBS, *gfp*, and B0015 terminator assembled in the DVK_AE vector from the CIDAR MoClo kit	[10,82]
3	TOPO-ß-carotene	ß-carotene in pCR™4Blunt-TOPO^®^	This study
4	pJV-ß-carotene	pJV298 with *crtY*, *crtB*, *crtI*, *crtE* under lacIq control	This study
5	TOPO-MVA1	MVA1 cluster in pCR™4Blunt-TOPO^®^	This study
6	TOPO-MVA2	MVA2 cluster in pCR™4Blunt-TOPO^®^	This study
7	pJV-ß-carotene-A5D5-MVA2	pJV-ß-carotene; J23102 promoter, B0032 RBS, *mvaK*, *mvaD*, *pmvk*, *idi*	This study
8	pJV-ß-carotene-MVA	pJV-ß-carotene; J23102 promoter, B0032 RBS, *thil*, *hmdH*, *hmcS*, MVA2	This study
9	Bba_J72214-BBa_J72090	P15A ori, CmR, araBAD promoter, *araC*, *vioA*, *vioB*, *vioC*, *vioE*	[43,83]
10	Synthesized *vioD* ^1^	Recoded; from *C. violaceum* ATCC 12472	This study
11	pVio	Bba_J72214-BBa_J72090 with *vioD*	This study

^1^ Synthesized by ATUM (https://www.atum.bio/).

**Table 3:** Primers used in this study.

#	Name	Sequence
1	CrtY_F	TCATCTCATCATTCCGATAGCGGCACT
2	CrtE_R	ATGAACAGTTCTTCTCGAAGTAAAGCCAG
3	pJV-ß-car-R	ATGTATATCTCCTTAAGCTTACGCC
4	pJV-ß-car-F	TGAGGATCCGGTGATTGATTG
5	Bcar-4pJV-F	gcttaaggagatatacatATGAACAGTTCTTCTCGAAG
6	Bcar-4pJV-R	aatcaccggatcctcaTCATCTCATCATTCCGATAG
7	Cluster 1 F	ATGAAAGATATTTATATTGTCGCTGC
8	Cluster 1 R	TTATTTAACTTTGTATTGACGAACATGGC
9	Cluster 2 F	ATGAAAAGTAGTTTTTTAGCTCATGG
10	Cluster 2 R	TTATTTAATTAACTGATCAATTTGATTTTTTAGTGGC
11	Clust1_ck-F	CGTTGTCGGTGGTTCGATTA
12	Clust1-ck-R	ACGAGCAACCCAACCTTATC
13	Clust2-ck-F	CTTAGGCGAACTGGCAGATATTA
14	Clust2-ck-R	TGAGGTTGGCACGTGATTAG
15	A5D5-F	tgccgctatcggaatgatgagatgaggagTTGACAGCTAGCTCAGTC
16	A5D5-R	gcagcgacaatataaatatctttcatcattTAGTACTTTCCTGTGTGACTC
17	Cluster2-SacI-R	actacttttcatgagctcTTATTTAACTTTGTATTGACGAACATG
18	Cluster2-SacI-F	caaagttaaataagagctcATGAAAAGTAGTTTTTTAGCTC
19	MVA2-ovrlp-R	caatcaatcaccggatcctcaTTATTTAATTAACTGATCAATTTGATTTTTTAG
20	pJV-bcar-n-R	TCATCTCATCATTCCGATAGCG
21	pJV-b-m2-4m1-F	CATGTTCGTCAATACAAAGTTAAATAAGAGC
22	pJV-b-m2-4m1-R	GCAGCGACAATATAAATATCTTTCATCAT
23	pJV-empty-F	GATCCGGTGATTGATTGAG
24	pJV-empty-R	CTTACATTAATTGCGTTGCGC
25	VioD-F	AAAATTCTGGTGATTGGCGCGGGC
26	VioD-R	ttaCTCGAGGCGCTGCAGCGC
27	Bba-4VioD-F	cgagtaaGGATCCGAGGCTTGGATTCTCA
28	Bba-4vioD-R	ttttttacctccttaaggatcTTAGCGCTTGGCCGCGAAA
29	vioD-inschk-F	GCGGTTTTCGCGGCCAAGCG
30	vioD-inschk-R	GGCAGGGCGGGGCGTAATTTGAT

Uppercase letters denote portion that anneals to original sequence.

**Table 4:** Selected DNA features.

Name	Sequence
P_tac_ promoter	ttgacaattaatcatcggctcgtataatg
J23102 promoter	ttgacagctagctcagtcctaggtactgtgctagc
B0032m RBS	agagtcacacaggaaagtacta

The MVA1 and MVA2 clusters were then inserted into the pJV-ß-carotene plasmid by Gibson assembly. First, primers 13 and 14 were used to PCR amplify the MVA2 cluster from plasmid TOPO-MVA2, and primers 15 and 16 were used to PCR amplify the J23102 constitutive promoter (from the Anderson promoter collection, http://parts.igem.org/Promoters/Catalog/Anderson) and B0032 RBS (the two together denoted as A5D5) from plasmid pA5D5GFP [10,82]. Primers 7 and 17 were used to PCR amplify pJV-ß-carotene. Assembly reactions were transformed into NEB10ß *E. coli* and produced the plasmid pJV-ß-carotene-A5D5-MVA2. The presence of MVA2 was confirmed by colony PCR (primers 13 and 14), plasmid restriction digestion mapping (*Nde*I), and by sequencing. 

To incorporate MVA1 into pJV-ß-carotene-A5D5-MVA2 between A5D5 and MVA2, a second Gibson assembly was performed. Primers 18 and 19 were used for MVA1 from TOPO-MVA1 and primers 4 and 20 were used for the vector. Since it has been recorded that products from the MVA1 cluster can accumulate and be toxic to cells [68], the reaction was transformed into NEB10ß *E. coli* and IPTG was added to the SOC recovery media to activate the beta-carotene cluster to attempt to pull the reaction towards product (~1 mM final). Recovery proceeded for 40 min. Outgrowth was plated on LB plates with IPTG at 1 mM final concentration, and incubated overnight at 37 °C. Colonies were selected for growth with IPTG and DNA preparation resulted in plasmid pJV-ß-carotene-MVA. The plasmid was confirmed by colony PCR with primers 11 and 12 for MVA1 and 13 and 14 for MVA2, plasmid restriction digestion mapping (*Spe*I and *Bbs*I, separately), and by sequencing. During sequencing, it was noted that a spontaneous mutation had occurred (G298T) in the *crtY* gene; however, HPLC analysis confirmed the strain still produced beta-carotene. Plasmid pJV-ß-carotene-MVA was then electroporated into *V. natriegens*, resulting in strain *V. natriegens* pJV-ß-carotene-MVA. For a list of DNA features used for the different plasmids, see Table 4.

### 4.5. Cloning of the Empty Control pJV298 Plasmid

To create an empty control vector variation of the pJV298 plasmid, we amplified the plasmid to exclude the region encompassing the lacIq repressor gene, promoter, and the Ptac promoter and *gfp* using the primers 23 and 24. The resultant PCR product was phosphorylated with NEB’s Polynucleotide Kinase (PNK) and then ligated using NEB’s T4 Ligase, according to manufacturer’s protocol. The resultant reaction was transformed into NEB10B cells according to manufacturer’s protocol. After overnight growth at 37 °C, colonies were analyzed for lack of green fluorescence upon IPTG induction (1 mM) overnight. For a list of DNA features used for the different plasmids, see Table 4.

### 4.6. Cloning of the Violacein Pathway

The plasmid containing most of the violacein pathway, BBa_J72214-BBa_J72090, was acquired from Addgene (BBa_J72214-BBa_J72090 was a gift from Christopher Anderson (Addgene plasmid # 40782; RRID:Addgene_40782; [43,83]). Information on the Addgene website indicated that sequencing confirmed the absence of the *vioD* gene [83]. Restriction enzyme digestion (*Sac*I-HF) suggested, and partial sequencing by the authors of this manuscript confirmed, the absence of *vioD*. A synthesized *vioD* (ATUM, Newark, CA, USA http://www.atum.bio) based on the sequence from *C. violaceum* ATCC 12472 was recoded for *E. coli* (similarly to the rest of the genes [43,44,83]) and inserted into the BBa_J72214-BBa_J72090 plasmid using Gibson Assembly. The *vioD* gene was amplified using primers 25 and 26, and the vector with primers 27 and 28. NEB10β cells were transformed with the assembly as recommended. Colonies were tested by colony PCR with primers 29 and 30. DNA preparation afforded the plasmid pVio, with insertion of *vioD* confirmed with partial sequencing at each end of the gene. Plasmid pVio was then electroporated into *V. natriegens*, resulting in strain *V. natriegens* pVio. It is noted that the encoded VioB in the plasmid from Addgene was a variant as compared to the natural enzyme from *C. violaceum* (S303N), but the cluster was still able to produce violacein. For a list of DNA features used for the different plasmids, see Table 4.

### 4.7. Heterologous Production of Beta-carotene

For production of beta-carotene in LBv2 rich medium, overnight cultures of *V. natriegens* transformed with pJV298-C (empty control plasmid), pJV-ß-carotene, and pJV-ß-carotene-MVA were each inoculated into triplicate shaker flasks containing 25 mL of LBv2 media supplemented with chloramphenicol (6 µg/mL final). Cultures were incubated at 30 °C (250 rpm) in the dark for ~2–3 h until cultures reached approximate mid-log growth phase (OD_600_ ~ 0.7–0.8), were induced with IPTG (~1 mM final), and incubated overnight (~19.5–20.3 h). An aliquot of 1.8 mL was centrifuged at 8000 rpm (~4720 rcf) for 3 min, the pellet washed with 500 µL of PBS supplemented with 300 mM NaCl, and centrifuged at 8000 rpm for 6 min. The pellet was dissolved in acetone and incubated at 55 °C in the dark for 15 min similar to a published procedure for extracting beta-carotene [26]. The solution was centrifuged at 16,000 rcf for 10 min and an 800 µL aliquot of supernatant removed, dried under reduced pressure, and stored at –80 °C until analysis.

For production of beta-carotene in minimal media supplemented with different carbon sources (0.4%), starter cultures of *V. natriegens* transformed with pJV-ß-carotene-MVA were made in each of the different media (supplemented with chloramphenicol at 6 µg/mL) and grown overnight at 30 °C (250 rpm). Triplicate production cultures (5 mL) of each media were inoculated with starter cultures, incubated at 30 °C (250 rpm) in the dark until cultures reached approximate mid-log growth phase (OD_600_ ~> 0.6), and then induced with IPTG (~1 mM final). Since cultures in different media grew at different rates, induction was staggered (1.5 h max time difference). After 19 h of induction, cultures were moved to 4 °C. Beta-carotene extraction was then done as described for rich medium growth.

Beta-carotene production was determined by HPLC-UV. Samples were dissolved in acetone, followed by addition of 70:30 acetonitrile: methanol for a final 2:1 ratio. Samples were then centrifuged at 16,000 rcf for 10 min at 4 °C, and a portion of supernatant transferred for analysis. Randomized samples were analyzed on a Waters Acquity H-Class UPLC with a 2.1 x 50 mm BEH C18 1.7 µm column (Waters, Milford, MA, USA) and beta-carotene detected using an in-line PDA eλ detector at 453 nm (2.4 nm resolution; integrated area), with mass/charge confirmation of select samples using an in-line Waters SQ Detector 2 mass spectrometer in ESI positive mode. Samples were compared to a standard curve from a ß-carotene standard (Sigma Aldrich, St. Louis, MO, USA). The analysis was conducted similarly to Paliakov et al. [84]. Buffer A was 90:10 acetonitrile:H_2_O and buffer B was 70:30 methanol:isopropanol, and samples (2 µL injections) were analyzed using the following gradient at 40 °C at a flow-rate of 0.4 mL/min: 0 min, 60% B; 1.25 min, 60% B; 2 min, 60–80% B (linear); 4.5 min, 80% B; 5.25 min, 80–60% B (linear); 7.25 min, 60% B; beta-carotene eluted at ~2.75 min. Data are the average of triplicates, and error represents the standard deviation of the mean.

### 4.8. Heterologous Production of Violacein

For production of violacein in LBv2 rich medium and minimal media supplemented with different carbon sources, starter cultures of WT *V. natriegens* and *V. natriegens* transformed with pVio were made in LBv2 and grown overnight at 37 °C (250 rpm). Duplicate production cultures (5 mL) of each medium (triplicate for LBv2) were inoculated with starter cultures, incubated at 37 °C (250 rpm) for 44 h, similar to the time-scale used in a previous publication [20]. An aliquot of 500 µL was centrifuged at 8000 rpm (≈ 4,720 rcf) for 3 min. The pellet was extracted twice with 250 µL ethanol × and a portion of this (300 µL) dried under reduced vacuum and stored at −20 °C until analysis. After extraction of the pellet once more with ethanol, the pellet was dissolved in PBS and an OD_600nm_ measurement taken to estimate cell density, similar to a published method [20].

Violacein production was determined by HPLC-UV. Samples were dissolved in ethanol, centrifuged at 16,000 rcf for 10 min at 4 °C, and 40 µL of supernatant transferred for analysis. Randomized samples were analyzed on the same instrumentation as for beta-carotene (see above) but the PDA detector was set at 575 nm and 560 nm for violacein and deoxyviolacein, respectively (both at 2.4 nm resolution). Samples were compared to a standard curve from a violacein standard (>98%, violacein from *Janthinobacterium lividum*, Sigma Aldrich, St. Louis, MO, USA) and a deoxyviolacein standard (deoxyviolacein, Cayman Chemical, Ann Arbor, MI, USA). Note that deoxyviolacein is contained within the violacein standard from Sigma Aldrich but appeared to be a minor constituent and so was not considered during standard curve calculation (sold as minimum 85% violacein). Buffer A was 0.1% formic acid in H_2_O and buffer B was 0.1% formic acid in acetonitrile, and samples (2 µL injections) were analyzed using the following gradient at 45 °C at a flow-rate of 0.4 mL/min: 0 min, 2% B; 2 min, 2% B; 7.5 min, 2–95% B (linear); 10 min, 95% B; 10.5 min, 95–2% B (instant); 12.5 min, 2% B; violacein eluted at ~4.64 min and deoxyviolacein eluted at ~5.04 min. Data are the average of duplicates (triplicate for LBv2 medium), and error for LBv2 medium represents the standard derivation of the mean.

### 4.9. Statistical Significance Analysis

For determination of statistical significance of beta-carotene production between *V. natriegens* expressing pJV-ß-carotene and pJV-ß-carotene-MVA in LBv2 medium, a two-sample assuming unequal variances t-test was done using Excel 2013 Data Analysis (Microsoft, Redmond, WA, USA). For determination of statistical significance of beta-carotene production or growth in minimal media supplemented with different carbon sources, first a single factor ANOVA was done using Excel 2013 Data Analysis (Microsoft, Redmond, WA, USA) to determine if the *p-*value was less than 0.05 and if *F* was greater than *F crit*. If so, a Tukey-Kramer analysis was done on each paired interaction, as calculated by: (1) determining the absolute difference, *d*, between averages calculated by ANOVA; (2) denoting the number of replicates, *n*, for each condition; (3) determining the standard error, SE, using the equation: SE = square root(0.5 × *MS_w_* × (1/*n*_1_ + 1/*n*_2_)), where *MSw* = the mean square error within groups calculated by ANOVA, and n_1_ and n_2_ are the biological replicates for each condition; and (4) determining the q statistic, *q*, as the absolute value of *d*/*SE* (Dr. Todd Grande, https://www.youtube.com/watch?v=_I4O3xxh2ns). Finally, *q* was compared to a table of *q* values (https://www2.stat.duke.edu/courses/Spring98/sta110c/qtable.html); if *q* was greater than the *q* value from the table for α = 0.05, *df* (as determined by ANOVA for within groups), and the number of conditions, the null hypothesis was rejected and the data was considered statistically different. 

## Figures and Tables

**Table 1:** Carbohydrates allowing *V. natriegens* cellular respiration.

Carbohydrates		
N-Acetyl-D-glucosamine ^1^	D-Galactose	Maltotriose
Adenosine	Gentiobiose	D-Mannitol ^1^
D-Arabinose	Glycerol ^1^	ß-Methyl-D-glucoside
L-Arabinose^1^	D,L-α-Glycerol phosphate	L-Rhamnose
D-Arabitol	D-Glucosamine	D-Ribose
Arbutin	α-D-Glucose ^1^	Sucrose^1^
D-Cellubiose	Inosine	Thymidine
2′-Deoxyadenosine	L-Lyxose	D-Trehalose
D-Fructose^1^	Maltose	Uridine

^1^ Denotes those selected for testing herein.

## Appendix A: Additional BioLog Results

**Table A1:** Carbon sources, categorized by BioLog (Hayward, CA), producing cellular respiration result above threshold level of 50 (*i.e.,* “positive”). Note that due to the proprietary nature of the experiment, comparing relative responses should be done with caution.

Compound	Response	Compound	Response
***Carbohydrates***		***Carboxylic acids, cont.***	
D-Glucosamine	159	Monomethyl succinate	82
D-Ribose	154	Butyric acid	81
Inosine	145	α-Ketobutyric acid	80
L-Arabinose	145	4-Hydroxybenzoic acid	73
Uridine	133	5-Keto-D-gluconic acid	70
2′-Deoxyadenosine	131	Formic acid	69
D-Fructose	131	Oxalomalic acid	54
Adenosine	128	D-Malic acid	50
D-Galactose	123	***Amino acids***	
Glycerol	122	Gly-Asp	149
ß-Methyl-D-glucoside	116	L-Glutamic acid	149
Thymidine	116	L-Asparagine	148
L-Rhamnose	106	L-Aspartic acid	148
D-Mannitol	105	D-Alanine	147
Arbutin	100	L-Glutamine	140
D-Cellobiose	95	Ala-Gly	137
Maltose	92	L-Proline	136
Maltotrioise	88	L-Serine	136
Sucrose	88	L-Threonine	129
α-D-Glucose	87	L-Alanine	128
D-Trehalose	82	Gly-Pro	117
N-Acetyl-D-Glucosamine	79	Hydroxy-L-proline	113
D-Arabinose	78	L-Histidine	108
Gentiobiose	73	L-Arginine	98
L-Lyxose	70	Glycine	96
D,L-α-Glycerol phosphate	65	Gly-Glu	94
D-Arabitol	56	L-Ornithine	80
***Carboxylic acids***		L-Pyroglutamic acid	79
L-Malic acid	145	D-Serine	52
Succinic acid	135	***Polymers***	
Fumaric acid	134	Dextrin	112
Pyruvic acid	132	Pectin	110
D-Gluconic acid	129	Laminarin	95
L-Lactic acid	128	Gamma-cyclodextrin	62
Acetic acid	119	***Fatty acids***	
D,L-Malic acid	119	Tween 20	109
Citric acid	116	Tween 40	108
Bromosuccinic acid	104	Tween 80	92
Propionic acid	102	***Esters***	
Malonic acid	97	Methylpyruvate	107
Quinic acid	97	***Amines***	
γ-amino-N-butyric acid	92	Putrescine	67
ß-hydroxybutyric acid	90	***Alcohols***	
α-Hydroxybutric acid	87	Dihydroxyacetone	58

## Appendix B: Additional Information Regarding Genes used in this Study

**Table A2:** Heterologous genes used in this report.

Protein	Gene	Gene ID	#bp ^3^	Notes ^4^	Source/Ref ^5^
Lycopene cyclase	*crtY* ^1^	5556089	1158	*crtB*(14)	Vc
Phytoene/squalene synthase family protein	*crtB* ^1^	5556092	915	*crtY*(14), *crtI*(20)	Vc
Phytoene desaturase	*crtI* ^1^	5556083	1614	*crtB*(20), *crtE*(11)	Vc
Polyprenyl synthetase	*crtE* ^1^	5556114	864	*crtI*(11)	Vc
Acetyl-CoA acetyltransferase	*thil*	3252766	1158(1169)	*hmdH*(1)	La
Hydroxymethyl glutaryl-CoA reductase	*hmdH*	3252708	1212	*thil*(1)	La
Hydroxymethyl glutaryl-CoA synthase	*hmcS*	3252698	1164		La
Mevalonate kinase	*mvaK*	3253078	909(923)		La
Mevalonate diphosphate decarboxylate	*mvaD*	3253057	963		La
Phosphomevalonate kinase	*pmvk* ^2^	3253097	1083(1097)		La
Isopentenyl pyrophosphate isomerase	*idi* ^2^	3253102	1020(1034)		La
L-tryptophan synthase	*vioA*	24947400	1257		Cv
Iminophenyl-pyruvate dimer synthase	*vioB*	24945600	2997		Cv
Protodeoxyviolaceinate monooxygenase	*vioE*	24949508	576		Cv
Tryptophan hydroxylase	*vioD*	24947515	1122		Cv
Violacein synthase	*vioC*	24948167	1290		Cv

^1^ Common name (homologous); ^2^ Common name (homologous) by searching protein name in UniProtKB; ^3^ base pairs (parenthesis include ribosome binding site sequence); ^4^ Overlaps with given gene (parenthesis indicate number of bp overlapping); ^5^ Gene source: Vc = *V. campbellii* BAA-1116 NCBI accession number NC_009783.1; La = *L. acidophilus* NCFM NCBI accession number NC_006814.3; Cv = *C. violaceum* ATCC 12472 NCBI accession number NC_005085.1.
